# Evaluating the Relationship of GDF-15 with Clinical Characteristics, Cardinal Features, and Survival in Multiple Myeloma

**DOI:** 10.1155/2020/5657864

**Published:** 2020-10-21

**Authors:** Małgorzata Banaszkiewicz, Jolanta Małyszko, Krzysztof Batko, Ewa Koc-Żórawska, Marcin Żórawski, Paulina Dumnicka, Artur Jurczyszyn, Karolina Woziwodzka, Joanna Tisończyk, Marcin Krzanowski, Jacek Małyszko, Anna Waszczuk-Gajda, Ryszard Drożdż, Marek Kuźniewski, Katarzyna Krzanowska

**Affiliations:** ^1^Chair and Department of Nephrology, Jagiellonian University Medical College, Kraków, Poland; ^2^Department of Nephrology, Dialysis and Internal Medicine, Medical University of Warsaw, Warsaw, Poland; ^3^Second Department of Nephrology and Hypertension with Dialysis Unit, Medical University of Bialystok, Bialystok, Poland; ^4^Department of Clinical Medicine, Medical University of Bialystok, Bialystok, Poland; ^5^Department of Medical Diagnostics, Jagiellonian University Medical College, Kraków, Poland; ^6^Chair and Department of Hematology, Jagiellonian University Medical College, Kraków, Poland; ^7^First Department of Nephrology and Transplantology with Dialysis Unit, Medical University of Bialystok, Bialystok, Poland; ^8^Department of Hematology, Oncology and Internal Diseases, Medical University of Warsaw, Warsaw, Poland

## Abstract

Growth differentiation factor 15 (GDF-15), a member of the transforming growth factor-*β* superfamily, participates in processes associated with myeloma development and its end-organ complications. It plays a significant role in both physiological and abnormal erythropoiesis and regulates iron homeostasis through modulation of hepcidin. It is abnormally secreted in marrow stromal cells of patients with multiple myeloma (MM), which may reflect the tumor microenvironment. We analyzed the associations of serum GDF-15 with clinical characteristics of 73 MM patients (including asymptomatic MM) and the laboratory indices of renal function, anemia, and inflammation. Baseline serum GDF-15 was studied as the predictor of two-year survival. We defined five clinically relevant subgroups of patients (symptomatic MM only, patients with and without remission, patients on chemotherapy, and without treatment). Increased GDF-15 concentrations were associated with more advanced MM stage, anemia, renal impairment (lower glomerular filtration and higher markers of tubular injury), and inflammation. Most of the results were confirmed in the subgroup analysis. Serum cystatin C and urine neutrophil gelatinase-associated lipocalin were associated with GDF-15 independently of other variables. In the studied MM patients, GDF-15 did not significantly predict survival (*p* = 0.06). Our results suggest that serum GDF-15 reflects myeloma burden and shares a relationship with several markers of prognostic significance, as well as major manifestations.

## 1. Introduction

Multiple myeloma (MM) is a common malignant condition resulting from a clonal proliferation of plasma cells in the bone marrow, which manifests itself with organ involvement, such as bone disease, anemia, and renal failure [1]. Unfortunately, despite several therapeutic advances introduced in recent years, MM remains an incurable condition for the majority of patients ([2, 3]; T. [4]). The disease microenvironment (ME) has become a focus of research, in which cellular interactions, growth factors, and cytokines have emerged as targets of interest [1]. Growth differentiation factor 15 (GDF-15; also known as macrophage inhibitory cytokine 1) has been recognized among the top interest cancer biomarkers [5]. The potential utility of GDF-15 in malignant neoplastic disease can be drawn from its unique characteristics of being a downstream target of tumor suppressor p53, with its only physiological presence at high levels in the placenta [6]. However, GDF-15 is also considered a divergent member of the transforming growth factor *β* (TGF*β*) family, which is induced in response to factors instigating cellular stress (e.g., hypoxia, tissue injury, and inflammation). These characteristics of GDF-15 suggest its increased concentration may be considered as an integrative, general marker for disease severity and mortality [6, 7].

Studies have attempted to elucidate the complex landscape of the myeloma ME. Schneiderova et al. [8] utilized an array of 92 cancer biomarkers among patients with overt myeloma and its premalignant stage, as well as an assessment following autologous stem cell transplant (auto-SCT). Prosurvival and chemoprotective factors were identified using highly sensitive immunoassays, among which GDF-15 was a prominent molecule, significantly enhanced in MM when comparing to monoclonal gammopathy of undetermined significance (MGUS). Westhrin et al. studied the potential of GDF-15, through its involvement with osteoclast differentiation, as a serum marker for bone lesions in MM [9]. Subsequent studies by Windrichova et al. utilized multiplex analytic technology to identify novel biomarkers for metastatic bone lesions, discerning GDF-15 as the most prominent biomarker [10]. It has been argued that improved understanding of the relationships between neoplastic plasmocytes and cells and factors constituting a disease-defining ME (interactions between the bone marrow and neoplastic cells, secretion of cytokines, survival, and growth factors) may elucidate myeloma pathophysiology, including mechanisms responsible for treatment failure [8].

Corre et al. (J. [11]) reported that among several established prognostic factors (including staging according to the International Staging System—ISS), in multivariate analysis, GDF-15 remained the only significant predictor of event-free survival, while in vitro experiments showed pretreatment with GDF-15 leads to improved survival of both stroma-dependent and independent MM cells when exposed to staple drugs in the MM treatment armamentarium (i.e., melphalan, bortezomib, lenalidomide). Furthermore, Mei et al. previously reported that both plasma GDF-15 concentrations and mRNA expression in peripheral blood mononuclear cells (PBMCs) were enhanced in ISS-III when comparing with stages I-II [12]. Taken together, the data presented in the literature support the relationship of GDF-15 with myeloma burden and disease progression.

Considering the attention that GDF-15 has received as a potential prognostic biomarker in neoplastic disease, we examined the serum concentrations of GDF-15 in an ambulatory population within the continuum of MM, ranging from asymptomatic up to overt MM (staged according to ISS). Our aim was to assess the relationships between the serum concentrations of GDF-15 and the disease characteristics defining the clinically relevant subgroups of patients. Moreover, we studied the correlations between GDF-15 and the acclaimed and emerging biomarkers of important clinical manifestations of MM, i.e., anemia and renal impairment. Finally, we searched for the association between serum GDF-15 and mortality in a heterogenous group of MM patients.

## 2. Materials and Methods

### 2.1. Study Design and Patients

Patients were recruited using convenience sampling during ambulatory control visits at the Departments of Hematology and Nephrology of the University Hospital in Kraków, Poland, between August 2016 and October 2017. The inclusion criteria were (1) age ≥ 18 years and (2) a diagnosis of SMM or MM according to the International Myeloma Working Group. Patients were excluded if any of the following were present: recent active infection; a history of hepatitis B, C, HIV; and neoplasms other than myeloma. Twenty-one healthy volunteers among physicians and medical staff (11 women, 10 men) aged between 24 and 69 years were recruited into the control sample. Physicians collected detailed history from all patients, which was supplemented by data from the available medical records. The data collected at the initial study visit included the age and sex, the date of initial diagnosis of SMM or MM, the current diagnosis, the results of bone imaging, and the information about past and present treatment including the response to treatment (CR, PR, SD, PD). In November 2018, the follow-up data were collected on mortality.

The study was conducted according to the principles of the Declaration of Helsinki and in compliance with the International Conference on Harmonization/Good Clinical Practice regulations. The study was approved by the Bioethics Committee of the Jagiellonian University, and all patients signed an informed consent for participation in the study.

### 2.2. Blood Samples and Laboratory Tests

Blood samples were obtained from patients at study inclusion. The blood samples of both patients and control subjects were collected in the morning following overnight fasting and rest. Routine laboratory tests were performed on the day of blood collection and included complete blood counts, serum concentrations of creatinine, iron, total protein, albumin, *β*2-microglobulin, free light chains, serum activity of lactate dehydrogenase, and urine concentrations of light chains.

In all patients, the aliquots of serum samples and urine samples were frozen and used for nonroutine laboratory tests, including the serum concentrations of GDF-15, IL-6, cystatin C, hepcidin, and N-terminal prohormone of brain natriuretic peptide and urine concentrations of NGAL monomer and cystatin C.

The Sysmex XE 2100 analyser (Sysmex, Kobe, Japan) was used for complete blood counts. The routine biochemical tests were carried out using automatic biochemical analysers: Hitachi 917 (Hitachi, Japan) and Modular P (Roche Diagnostics, Mannheim, Germany). The eGFR was calculated based on serum creatinine using the Modification of Diet in Renal Disease (MDRD) formula: eGFR = (186 × serum creatinine (*μ*mol/l) × 0.0113)^−1.154^ × age^−0.203^ × 114 (×0.742 for women). The concentration of serum FLC, urine LC (*κ* and *λ* type), and *β*2-microglobulin were measured by the immunonephelometric method on a BN II analyser (Siemens GmbH, Germany). The determination of free light chains (FLC Κ, FLC Λ) was performed using Freelite reagents (Binding Site, Birmingham, UK) with reference ranges of 1.7-3.7 g/l and 0.9-2.1 g/l, respectively. The immunophenotype of monoclonal protein was determined by serum immunofixation (IFE) on agarose gel (EasyFix G26, Interlab, Italy).

The nonroutine laboratory tests were performed in series, using commercially available immunoenzymatic test kits. Serum IL-6 was measured using the Quantikine ELISA Human IL-6 Immunoassay (R&D Systems, Inc., Minneapolis, USA), with the minimum detectable dose of 0.70 pg/ml and the intra- and interassay precision of 2.0% and 3.8%, respectively. The reference range for IL-6 was 3.13–12.5 pg/ml. Serum GDF-15 was measured using the Quantikine ELISA Human GDF-15 Immunoassay (R&D Systems, Inc., Minneapolis, USA), with the minimum detectable dose ranging from 0.0 to 4.4 pg/ml and the intra- and interassay precision of 2.8% and 5.6%, respectively. The reference range for GDF-15 was 337–1060 pg/ml. Serum hepcidin 25 levels were measured using the Hepcidin 25 human Cet. No. S-1337 kit (Peninsula Laboratories International, Inc., San Carlos, USA). The reference range for hepcidin 25 is 0.02-25 pg/ml. Urine NGAL monomer was assessed using the Human NGAL monomer-specific ELISA kit (BioPorto Diagnostics A/S, Hellerup, Denmark), with the minimum detectable dose of 10 pg/ml. The detection range for NGAL 10-1000 pg/ml. NT-pBNP concentrations in serum were measured by the Enzyme-linked Immunosorbent Assay Kit For NT-ProBNP Human (Cloud-Clone Corporation, Huston, USA), with the minimum detectable dose of 11.7 pg/ml, the quantification range of 30.9-2.500 pg/ml, and the intra- and interassay precision of 10% and 12%, respectively. Cystatin C concentrations in urine and serum were measured using Human Cystatin C ELISA (BioVendor Research and Diagnostic Products, Brno, Czech Republic), with the detection range of 0.25–25 ng/ml and the intra- and interassay precision of 3.5% and 10.4%, respectively.

This study was conducted with the use of the equipment purchased by the Medical University of Bialystok as part of the RPOWP 2007-2013 funding, Priority I, Axis 1.1, contract No. UDA-RPPD.01.01.00-20-001/15-00 dated 26.06.2015.

### 2.3. Statistical Analysis

The number of patients and the percentage of the studied group were reported for categories. Mean ± standard deviation was reported for normally distributed and median (lower-upper quartile) for nonnormally distributed quantitative variables. Shapiro-Wilk's test was used to assess normality. The GDF-15 concentrations (a nonnormally distributed variable) were compared between subgroups using Mann-Whitney's test (two subgroups) or Kruskal-Wallis ANOVA (more than two subgroups). The boxplots illustrating the differences between subgroups show the median (central line), interquartile range (box), nonoutlier range (whiskers), and outliers (points) in each subgroup. The Spearman rank correlation coefficient was used to assess simple correlations of GDF-15. Right-skewed variables were transformed using normal logarithm (ln) to increase the readability of the scatterplots illustrating the simple correlations. Also, ln-transformed right-skewed variables were used to calculate linear regression models presented in Table 1. Simple and multiple Cox proportional hazard regression was used to study the predictors of overall mortality. In general, the regression models (linear and Cox) were constructed using predictor variables that were significantly associated with the outcome variable in the simple analysis. In case of multiple intercorrelated predictor variables (linear regression), we chose the ones that were most strongly correlated with the dependent variable. The statistical tests were two-tailed, and *p* < 0.05 indicated statistical significance. Statistica 12.0 (StatSoft, Tulsa, USA) was used for computations.

## 3. Results

### 3.1. Demographic and Clinical Characteristics of the Studied Patients with Multiple Myeloma

The prospective study recruited patients diagnosed with MM, assessed during their control ambulatory visits between August 2016 and October 2017. Seventy-three patients with MM (35 women, 38 men) aged between 29 and 90 years were enrolled in the study (Table 2). Smoldering myeloma (SMM) was diagnosed in six patients and MM in 67 patients. Most patients were in stage I according to the International Staging System (ISS) (Table 2) [13]. Except for 8 patients (including the subjects diagnosed with SMM), all patients underwent at least one line of treatment, and over one-third underwent autologous bone marrow transplantation before entering the study. At the start of the study, roughly 70% of patients were in complete or partial remission (Table 2).

Bone lesions were observed in 60% of patients (Table 2). Six patients experienced acute kidney injury prior to enrollment, and the estimated glomerular filtration rate (*e*GFR) below 60 ml/min/1.73 m^2^ was observed in 26 patients (36%) at recruitment (Table 3). Nearly one-fifth of the patients had low hemoglobin concentrations at the start of the study (Table 3).

### 3.2. Circulating GDF-15 Is Elevated in Multiple Myeloma and Associated with ISS Stage

Twenty-one healthy volunteers matched with the patients according to sex (8 women, 13 men; *p* = 0.3 versus studied group) and age range (39 to 66 years) were recruited to provide blood samples in order to obtain reference values for nonroutine biomarker assays. Aside from GDF-15 and IL-6, the remaining markers (Table 4) were not significantly altered in MM patients when compared to healthy reference subjects. Although the mean age of controls (51 ± 9 years) was lower compared to the studied group (*p* < 0.001); the differences in GDF-15 (*p* = 0.018) and interleukin 6 (*p* = 0.049) concentrations between MM patients and controls were independent of age.

**Figure 1:**
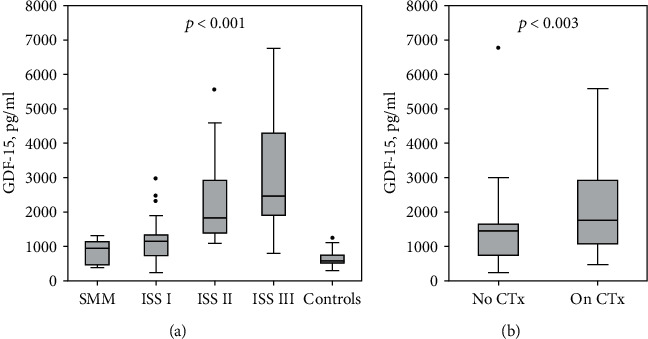
Serum GDF-15 concentrations according to incremental MM disease stages with reference controls (a) and among patients receiving no chemotherapy (CTx) treatment versus those on chemotherapy at baseline (b). Data are shown as median (horizontal line), interquartile range (box), nonoutlier range (whiskers), and outliers (points); *p* value is shown for overall comparison between groups. The numbers of patients in each group are reported in Table 2. SMM: smoldering myeloma; ISS: International Staging System for multiple myeloma.

The serum concentrations of GDF-15 were positively correlated with the ISS stage (*R* = 0.65; *p* < 0.001) and differed significantly between patients across stages of MM (Figure 1(a)). In post hoc comparisons, patients with ISS stage II or III differed significantly from those with smoldering myeloma or stage I MM, and only patients with smoldering MM did not differ significantly from controls. Patients with complete remission (CR) had lower GDF-15 concentrations than the rest of the group: the median (lower; upper quartile) of GDF-15 was 1029 (758; 1402) pg/ml in CR versus 1390 (925; 2466) pg/ml in the remaining patients (*p* = 0.013). However, there were no significant differences regarding the GDF-15 concentrations between MM patients with partial remission (PR), stable disease (SD), or progressive disease (PD): 1545 (1014; 2216) pg/ml in PR; 910 (653; 3018) pg/ml in SD, and 1681 (1168; 2921) in PD (*p* = 0.7). No statistically significant correlations were observed between circulating GDF-15 and age (*R* = 0.22; *p* = 0.065) as well as time from MM diagnosis (*R* = −0.16; *p* = 0.2). The patients who received maintenance chemotherapy treatment at the start of the study had higher circulating GDF-15 levels compared to those without treatment (Figure 1(b)); however, complete remission was more common in untreated patients (44% versus 10% among those receiving treatment; *p* = 0.002). The details regarding treatment regimens (Table S1) and GDF-15 concentrations according to drugs used (Figure S1) are shown in Supplementary File.

**Figure 2:**
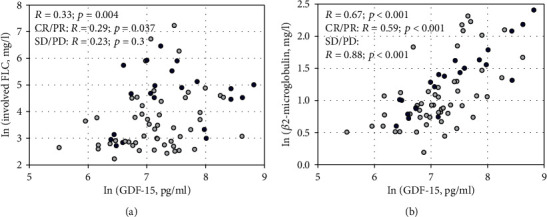
Correlations between serum GDF-15 concentrations and involved serum free light chains (FLC) (a) and *β*2-microglobulin (b) in studied patients. Light circles represent patients with complete or partial remission (CR/PR), and dark circles represent patients without remission (stable disease or progressive disease, SD/PD). The right-skewed variables were transformed using the natural logarithm (ln) to enhance readability. The Spearman rank-order correlation coefficients (*R*) in the whole studied group and the subgroups are shown with associated *p* values.

In MM patients, serum GDF-15 positively correlated with the concentrations of involved (i.e., *κ* in patients with monoclonal protein including *κ*-type light chains and *λ* in patients with *λ*-type monoclonal protein) free light chains in serum (*R* = 0.33; *p* = 0.004; Figure 2(a)) and involved light chains in urine (*R* = 0.35; *p* = 0.002) as well as with total (*κ* plus *λ*) serum free light chains (*R* = 0.35; *p* = 0.002) and urine light chains (*R* = 0.37; *p* = 0.001). Moreover, GDF-15 concentrations were highly positively correlated with *β*2-microglobulin (*R* = 0.67; *p* < 0.001; Figure 2(b)) and negatively with serum albumin (*R* = −0.52; *p* < 0.001). We defined five subgroups of patients according to important clinical features as recorded at the start of the study: (1) patients with symptomatic MM (i.e., after excluding those with SMM) (*n* = 67), (2) patients in remission (CR/PR) (*n* = 52) and (3) those without remission (SD/PD) (*n* = 21), and (4) patients receiving maintenance treatment (*n* = 30) and (5) not receiving chemotherapy (*n* = 43). In all subgroups, we confirmed positive correlations between GDF-15 and *β*2-microglobulin, and negative correlations between GDF-15 and albumin. Free light chains in serum and light chains in urine correlated with GDF-15 in patients with symptomatic MM, in remission, and those not receiving chemotherapy (Table S2, Supplementary File).

### 3.3. Circulating GDF-15 Is Associated with Anemia in Multiple Myeloma

**Figure 3:**
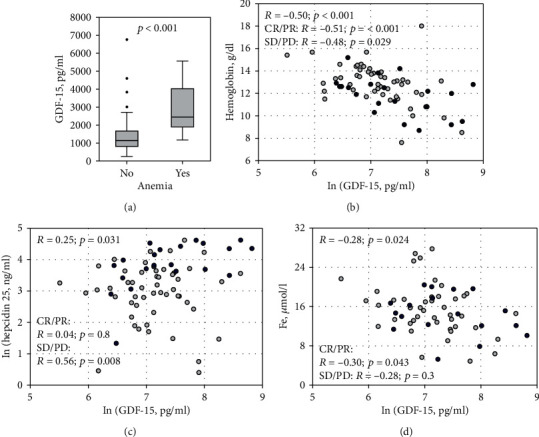
Serum GDF-15 concentrations among patients with (*n* = 14) and without (*n* = 59) anemia (a). Data are shown as median (horizontal line), interquartile range (box), nonoutlier range (whiskers), and outliers (points). Correlations between serum GDF-15 and blood hemoglobin (b), serum hepcidin 25 (c), and iron (d) in the studied patients. Light circles represent patients with complete or partial remission (CR/PR) and dark circles represent patients without remission (stable disease or progressive disease, SD/PD). The right-skewed variables were transformed using natural logarithm (ln) to enhance readability. The Spearman rank-order correlation coefficients (*R*) in the whole studied group and the subgroups are shown with associated *p* values.

Serum GDF-15 concentrations were significantly higher in the studied patients with anemia and inversely correlated with blood hemoglobin and serum iron (Figures 3(a), 3(b), and 3(d)). The association between GDF-15 and blood hemoglobin was significant in all studied subgroups. Moreover, the association between GDF-15 and iron was confirmed in subgroup analyses: after excluding patients with SMM, in patients on chemotherapy and in patients with complete or partial remission. In the study group overall, a weak positive correlation was found between GDF-15 and serum hepcidin-25 (Figure 3(c)). In MM patients without remission (SD/PD), this association was much stronger (*R* = 0.56; *p* = 0.008), while in other subgroups, it was nonsignificant.

### 3.4. Circulating GDF-15 Is Associated with Renal Function in Multiple Myeloma

**Figure 4:**
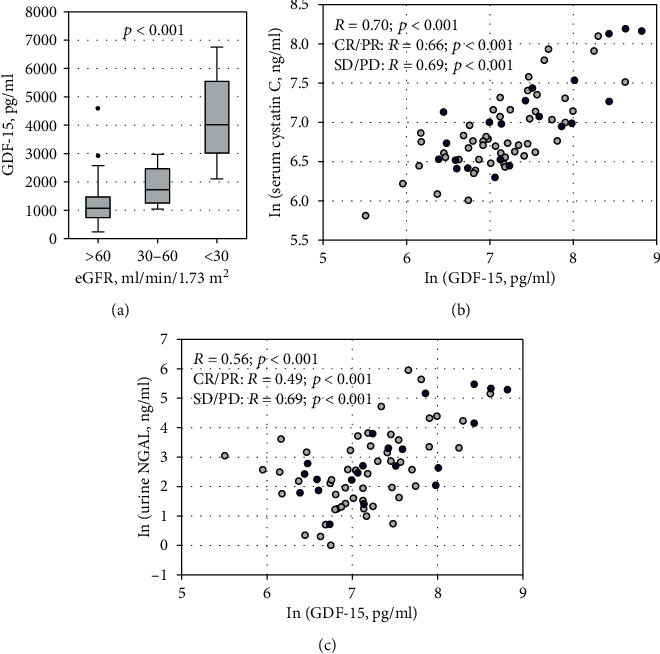
Serum GDF-15 concentrations among studied patients according to eGFR values: >60 ml/min/1.73 m^2^ (*n* = 47), 30-60 ml/min/1.73 m^2^ (*n* = 17), and <30 ml/min/1.73 m^2^ (*n* = 9) (a). Data are shown as median (horizontal line), interquartile range (box), nonoutlier range (whiskers), and outliers (points). Correlations between serum GDF-15 and cystatin C (b) and urine NGAL (c). Light circles represent patients with remission, and dark circles represent patients without remission. The right-skewed variables were transformed using natural logarithm (ln) to enhance readability. Spearman rank-order correlation coefficients (*R*) in the whole studied group and the subgroups are shown with associated *p* values.

Higher GDF-15 concentrations were observed in patients with eGFR < 60 ml/min/1.73m^2^ as compared to patients with better kidney function (*p* < 0.001; Figure 4(a)). Highly significant positive correlations were observed between serum GDF-15 and markers of glomerular filtration: serum creatinine (*R* = 0.57; *p* < 0.001) and serum cystatin C (*R* = 0.70; *p* < 0.001; Figure 4(b)). Consequently, negative correlations were observed between GDF-15 and eGFR values based on these markers (*R* = −0.54, *p* < 0.001; and *R* = −0.67, *p* < 0.001, respectively). These associations were confirmed in all studied subgroups of patients (after exclusion of SMM, with and without remission, on chemotherapy, and without chemotherapy). Moreover, in the study group overall, GDF-15 concentrations positively correlated with the studied markers of tubular injury: urine NGAL monomer (Figure 4(c)) and urine cystatin C (*R* = 0.24; *p* = 0.040), although the latter correlation was weak. Of note, urinary NGAL (*R* = −0.31; *p* = 0.008) but not urinary cystatin C was significantly correlated with eGFR. The association between GDF-15 and urine NGAL was significant in every subgroup, while the correlation between GDF-15 and urine cystatin C was only significant in patients without remission (SD/PD) (*R* = 0.51; *p* = 0.016).

### 3.5. Other Variables Significantly Associated with Circulating GDF-15 in MM and Multiple Regression

In the studied group, weak positive correlations were observed between the marker of systemic inflammation, interleukin 6 (IL-6), and GDF-15 (*R* = 0.31; *p* = 0.007). Interestingly, the total white blood cell counts and GDF-15 (*R* = 0.27; *p* = 0.022) were significantly correlated. Both correlations were also significant after excluding subjects with SMM; moreover, GDF-15 was significantly correlated with IL-6 in subjects without remission and those not on chemotherapy.

Circulating GDF-15 was weakly positively correlated with serum NT-proBNP (*R* = 0.28; *p* = 0.016) in the whole studied group and after the exclusion of SMM patients.

Using the data of all the studied patients, we performed multiple regression analysis in order to assess which variables are associated with GDF-15 concentrations independently of each other (Table 1). Firstly, among the clinical characteristics describing MM state which were associated with GDF-15 in simple analysis, the ISS stage together with chemotherapy treatment status was identified as independent predictors of GDF-15 (Table 1, model 1). Secondly, laboratory data were added to these predictors, and the markers of kidney function, i.e., serum cystatin C and urine NGAL monomer, were identified as significant predictors of serum GDF-15, independent of ISS, treatment status, hemoglobin, and interleukin 6 (Table 1, model 2).

### 3.6. Association of GDF-15 with Mortality

In November 2018, the follow-up data on all-cause mortality were collected. The median (lower; upper quartile) observation time in the studied group was 20 (16; 23) months, range from 1 to 25 months. During the observation period, 15 (21%) patients died. Two-year survival (calculated from recruitment into the study until death or the end of follow-up) was 79%. ISS stage (II or III versus I) and positive chemotherapy status on recruitment were identified as significant predictors of worse survival. GDF-15 (ln-transformed) was not significantly associated with overall survival, neither in simple analysis nor after adjustment for these predictors; however, the association was close to being statistically significant (Table 5). Notably, the direction of this association changed from negative in the simple analysis (HR higher than 1) to positive (HR below 1) after adjustment for the powerful predictors of survival, i.e., ISS and treatment status.

## 4. Discussion

Renal failure, which is present in approximately 30% of newly diagnosed MM (NDMM) patients, is a major cause of early mortality, and an independent poor prognostic factor of survival. However, renal impairment (RI) is potentially reversible in up to 50% of patients, which may improve long-term outcomes [14–16]. In the present study, circulating GDF-15 was highly correlated with markers of glomerular filtration (serum creatinine and cystatin C) as well as markers of tubular injury (urinary NGAL and cystatin C). Our salient finding is that serum cystatin C and urinary NGAL were the strongest and independent predictors of serum GDF-15. This could indicate a close link between the mechanisms underlying MM progression and renal complications.

Renal injury occurs from a variety of pathological mechanisms associated with the solubility and nephrotoxic profile of the circulating immunoglobulins and paraproteins, which may precipitate and form casts in distal tubules or induce a proinflammatory and profibrotic milieu when excess light chain endocytosis occurs [17, 18]. Lipocalin 2 (NGAL), an acute-phase molecule with iron-chelating features, characterized with sensitivity to early kidney damage (particularly of tubular origin), and cystatin C, a freely filtered, proximally absorbed, and catabolized, nonsecreted protein, have both been identified as novel markers of RI in MM [19]. Potential utility in very early detection of RI may translate into prompt treatment and renal outcome improvement, though this has not yet been well studied [20–22]. It is noteworthy to consider the “Forest Fire Theory” of Mori and Nakano, which proposes NGAL as a marker indicative of real-time kidney damage, derived from continuous production by inflamed tubular cells, rather than as a consequence of nephron loss (which would be more accurately reflected in cystatin C concentrations) [23]. Moreover, plasma and urinary sources of NGAL may differ; injury in distal nephron segments leads to secretion into the urine, while plasma concentrations could also be derived from nonrenal sources (e.g., increased in inflammatory states) [24]. A response to kidney insult, rather than inflammation, seems to be the major inducer of NGAL in MM, while a rise in both urinary NGAL and serum cystatin C may be indicative of a tubular-glomerular axis impairment, for which these molecules are respective markers [20]. Moreover, serum cystatin C levels have been noticed to be an independent prognostic factor for survival and correlate with advanced ISS stage [22]. Overall, we observed a strong relationship between novel indices of kidney injury and GDF-15. This may suggest that GDF-15 shares a close relationship with ongoing and/or permanent renal damage. In our analysis of patients on maintenance treatment, the trends between GDF-15 and markers of injury to kidney compartments were of strong significance and were less pronounced in the group of nontreated patients, where remission was more common. Whether GDF-15 is a key player in the processes shaping kidney injury or it is intertwined with myeloma progression and resulting end-organ sequelae remains to be established.

GDF-15 has been shown to increase the survival of stroma-dependent MM cells, though it is not produced by the neoplastic cells themselves, but rather by bone marrow (BM) mesenchymal stem cells. These stromal cells have been documented with an abnormal gene expression profile (termed “tumor ME genes”), which seems to support the malignant clone and promote its proliferation and survival (J [25]; J. [11]). Subsequent studies have shown that BM stromal cells produce GDF-15 after direct contact with tumor cells, while GDF-15 strongly enhances their clonogenic growth and self-renewal (T. [4]). Earlier studies in newly diagnosed myeloma patients have established GDF-15 as a promising molecule of prognostic significance, with the potential to identify patients with poor response to therapy and worse prognosis (J. [11, 26]). Our study examined an ambulatory care sample of patients with MM, demonstrating that serum GDF-15 concentrations are significantly associated with myeloma characteristics, even in a diverse clinical population at various stages of disease (though the majority was in stage I according to ISS). This is supported by the relationships apparent across different patient subgroups (remission, stable, and progressive disease), which suggests that GDF-15 may be useful not only in the treatment-naive patients but as an additional indicator of tumor burden, which we postulate to reflect the underlying myeloma microenvironment. Patients subjected to the maintenance chemotherapy have higher GDF-15 concentrations, with complete remission less common in these patients (10% vs. 44%), which may be explained by residual disease as a driver of higher circulating GDF-15 concentrations. Indeed, the differences in GDF-15 concentrations across different study groups were only evident when comparing complete remission with partial remission, stable, and progressive disease (significantly lower only in CR). This may indicate that in cases of partial vs. complete remission, the tumor microenvironment exerts a protective effect on myeloma cells, which is reflected in circulating GDF-15 concentrations similar to stable or progressive disease. GDF-15 may be a valuable molecule to investigate in biomarker-based models accounting for the complexity of myeloma biology. The characterization of myeloma risk and the patient phenotype, i.e., whether they will benefit from a particular therapeutic strategy, is an important and pragmatic aspect of management [27]. However, cytogenetic and molecular characterization is not readily available or accessible in all centers; therefore, a relevant model of disease-based biomarkers is of relevance for routine practice.

A prior analysis of a group of 15 patients with MM showed that significantly longer progression-free survival was present in patients with decreasing GDF-15 levels (T. [4]). Similarly, other studies support the relationship between GDF-15 and prognostic significance, but the association with survival is controversial. The research by the team of Corre et al. showed that lower plasma GDF-15 is associated with better event-free and overall survival, though this was not confirmed in subsequent studies (J. [11, 26]). However, in the study by Westhrin et al., lower serum GDF15 was associated with improved survival, more advanced osteolytic bone disease, and a relationship with serum measures of osteoclast activity. Further in vitro experiments showed promotion of osteoclast differentiation and osteoblast inhibition, which together supports the role of GDF15 in another cardinal myeloma feature—bone disease [9]. We were not able to confirm a significant association between circulating GDF-15 levels and overall two-year survival in regression models, and remarkably, the direction of the trend was reversed after accounting for disease stage and chemotherapy, which were strong determinants of poor survival. GDF-15 may therefore be a molecule reflecting myeloma burden as a consequence of the progressive disease, and therefore, its assessment throughout the management does not hold more favorable prognostic significance to the staging system itself.

Following the indications from pathogenesis, we evaluated whether GDF-15 shares a relationship with biomarkers involved in the development of its cardinal features (hepcidin as a regulatory molecule of anemia of chronic disease, IL-6 as a major myeloma cytokine, and novel molecules of tubular and glomerular injury). Correlations between markers of functional nephron loss (cystatin C, eGFR formulas) and active tubular injury (urinary NGAL) were much more pronounced in patients necessitating treatment (i.e., maintenance chemotherapy), which may indicate that circulating GDF-15 reflects the symptomatic character of diseases. BM stromal cells have been reported as a major source of interleukin 6, which is a key player of inflammatory pathways in myeloma [28, 29] and inducer of hepcidin [30], which further emphasizes the importance of the abnormal ME. Alongside the concept of BM “crowding out” by tumor cells, it has been demonstrated that hematopoietic stem and progenitor cells (particularly erythroid precursors) may be reduced from the functional impairment, which has been tied to the adverse tumor ME, and particularly TGF*β* signaling [31]. GDF-15 may be a mediator of the tumor ME (J [25]; J. [11]).

The cellular targets and biological functions of GDF-15 are still being explored. The recent discovery of the brainstem-restricted receptor GFRAL [32] supports a primary role of GDF-15 as a signal of somatic distress for the central nervous system (CNS), with a regulatory effect on metabolic and anorectic activity [33]. GDF-15 also plays a part in the inflammatory response, and the work by the group of Luan et al. showed a GFRAL-related central metabolic adaptation via the regulation of sympathetic outflow and triglyceride metabolism [34]. Importantly, it has also been emphasized that the manifold actions ascribed to GDF-15 in prior research are limited by contamination of recombinant GDF-15 with TGF*β* [33, 35, 36]. This indicates that the previous implication of GDF-15 effects on Smad2/3 activity or TGF*β*RII requires caution and reappraisal [36]. On the other hand, it should also be noted that GDF-15 exerts direct effects on immune cells and its immunomodulatory capacity may be independent of centrally regulated mechanisms [36]. Further understanding of whether and if the central (GFRAL) and peripheral (local, fast-acting) GDF-15-associated mechanisms exist and interact with myeloma biology is warranted.

These studies provide a preliminary rationale for studying GDF-15 as a purported marker of the tumor ME in MM. Our findings revealed a strong relationship between serum GDF-15 concentrations and the disease spectrum (ranging from SMM to ISS stage III MM), as well as correlations between circulating GDF-15 and indices of tumor burden (albumin, *β*2-microglobulin), which fall in line with earlier reports ([26]; J. [11]). Patients in complete remission had significantly lower levels of GDF-15 when comparing with stable or progressive disease. However, these differences were marginally significant, which may also reflect an insufficient sample among subgroups.

Recombinant GDF-15 has been reported to inhibit hematopoiesis, which falls in line with negative correlations between hemoglobin and increasing levels of GDF-15 observed at present, as well as in prior works (Toshihiko [37]; Jill Corre, Hébraud, and Bourin 2013; J. [11]). The relationship between GDF-15 and markers of impaired hematopoiesis was confirmed in all studied subgroups, which confirms and extends the results of earlier studies. Moreover, the association of hepcidin with GDF-15, which may act as a regulatory molecule for the latter, is more pronounced in patients with stable or progressive disease, while the trend loses significance in remission. On the other hand, increased GDF-15 concentrations may also reflect more advanced or aggressive disease, which may more often lead to bone marrow impairment, and thus explain an association with lower hemoglobin levels. Whether the purported roles of GDF-15 remain independent of TGF*β* activity (with respect to contamination of recombinant GDF) or are invalidated remains to be confirmed. The ability of GDF-15 to regulate erythropoiesis through the suppression of hepcidin, the main regulator of iron metabolism, has prompted further investigations, which have implied an alternating role of GDF-15 in the context of underlying stimuli (primary disease, inflammatory milieu, and status of iron metabolism) [38]. The liver produces hepcidin in response to inflammatory cytokines, such as interleukin 6, though this process is also influenced by iron availability, hypoxia, and erythropoietin (EPO) [38]. In previously untreated MM patients, or those not in remission, hepcidin and GDF-15 are overexpressed in peripheral blood mononuclear cells, with plasma concentrations of GDF-15, interleukin 6, EPO, and hepcidin noticeably elevated [12]. We observed that our patients had elevated levels of interleukin-6, suggesting an underlying, prevalent state of inflammation, which may have modestly elevated circulating GDF-15 levels. As opposed to the inhibitory effect associated with very high GDF-15 concentrations, moderate elevation may stimulate hepcidin and explain the positive relationship noted at present (Toshihiko [37]).

Induction of hepcidin by GDF-15 may potentially limit the availability of iron for hematopoiesis and provide an alternative explanation for the relationship with anemia. It should be noted that the majority of our patients achieved PR/CR and were subjected to treatment, likely decreasing the levels of hepcidin and GDF-15, which falls in line with the observations from earlier studies [12]. Małyszko et al. previously examined the relationship between iron homeostasis and GDF-15 in kidney allograft recipients showing a significant elevation of GDF-15 in patients with anemia, while concentrations of GDF-15 and hepcidin, an independent predictor of GDF-15, were raised overall [39].

**Figure 5:**
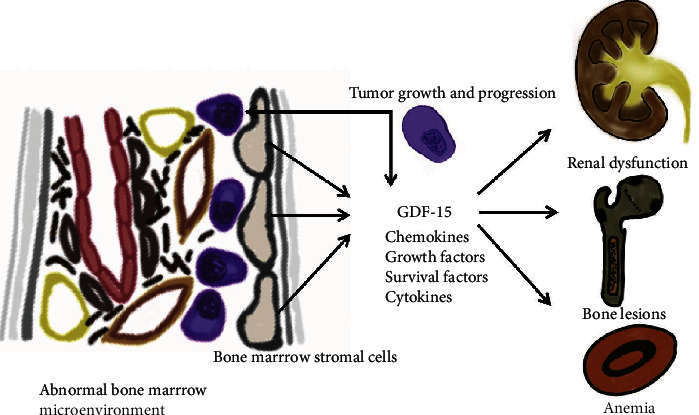
The proposed graphical representation of the bone marrow environment in multiple myeloma and end-organ involvement: abnormal bone marrow stromal cells release growth factors, cytokines, and survival factors (including GDF-15) following interactions with malignant plasma cells, which leads to uncontrolled tumor growth and progression. GDF-15 is associated with the development of the cardinal features of myeloma (anemia, renal impairment, and bone lesions), and it may be a mediator of pathways related to the development and progression of these complications.

In elderly individuals with anemia of unknown etiology, the moderate elevation of GDF-15 was observed, the latter being strongly correlated with kidney function (creatinine), which was surmised to indicate a mutual relationship between anemia and renal insufficiency [40]. Research has revealed that intrarenal expression of GDF-15 in the tubulointerstitial compartment correlates with its circulating levels, for which an increase in the latter may independently reflect renal function deterioration [41]. Large studies of the Framingham community have shown that GDF-15 is associated with new-onset renal disease and the decline in kidney function [42]. In AL amyloidosis, serum GDF-15 was recently revealed as the most significant prognostic measure for dialysis and a valuable addition to renal risk stratification [43]. The underlying mechanism of GDF-15 in both MM- and AL-related renal pathology is unclear, though in AL-amyloidosis, it has been suggested that GDF-15 plays a direct role in pathophysiology, and its production may be induced following a response to free light chain toxicity [43]. Similarly, we show that circulating GDF-15 correlates with the involved monoclonal light chains, and there is a significant relationship with involved paraproteins in urine or serum of patients who are not subject to treatment. GDF-15 seems to be an integral component of the tumor ME, for which it may serve as a surrogate measure, alongside other indices characterizing disease burden. The aforementioned studies suggest that GDF-15 may also be a mediator of processes involved in developing anemia and renal dysfunction, for which an indirect measure could be attained by assaying circulating forms, potentially holding diagnostic and prognostic significance (graphical presentation in Figure 5). However, it should be emphasized that the increased understanding of the molecular mechanisms of GDF-15 activity, particularly with regard to central GFRAL activity, is necessary to establish future utility.

### 4.1. Limitations

There are several weaknesses of the study that have to be emphasized. Due to this study being a single-center investigation, we recruited a heterogenous population in favor of study sample size, which prompted post hoc analysis to delineate patient subgroups of clinical interest. Prior studies in homogenous samples (i.e., incident myeloma cases) have already demonstrated that GDF-15 is a candidate marker in myeloma, yet the majority of patients that stand to benefit from biomarker assays throughout the management are not treatment-naive.

## 5. Conclusions

We postulate that GDF-15 may parallel the underlying mechanisms of myeloma pathology occurring in the abnormal ME, and as such, it shares a relationship with tumor burden and disease complications, most prominently related to anemia and kidney injury. Due to the correlations between GDF-15 and other markers of myeloma burden, which are also indicators of renal injury (e.g., *β*2 microglobulin), it is also conceivable that the pronounced concentrations of GDF-15 apparent at more advanced stages of disease relate to impaired glomerular filtration. However, the relationship with urinary NGAL in all clinical subgroups, which is reflective of active inflammation in the tubular compartment, may suggest that a generalized insult (e.g., cytokine byproducts of tumor progression) is more mechanistically related to the circulating levels of GDF-15, rather than functional nephron loss.

## Figures and Tables

**Table 1:** Multiple regression models showing the independent predictors of serum GDF-15 among the studied patients with MM. The right-skewed variables (including GDF-15) were ln-transformed before analysis.

Independent variable	Model 1		Model 2	
	Standardized regression coefficient ± standard error	*p* value	Standardized regression coefficient ± standard error	*p* value
SMM	−0.06 ± 0.09	0.5	Not included	-
ISS II	0.42 ± 0.10	<0.001	0.13 ± 0.09	0.1
ISS III	0.55 ± 0.09	<0.001	0.13 ± 0.09	0.2
SD/PD	−0.06 ± 0.10	0.5	Not included	-
On CTx treatment	0.25 ± 0.10	0.012	0.13 ± 0.07	0.07
Hemoglobin	Not included	-	−0.11 ± 0.08	0.2
ln (interleukin 6)	Not included	-	0.14 ± 0.08	0.08
ln (cystatin C)	Not included	-	0.43 ± 0.11	<0.001
ln (uNGAL)	Not included	-	0.25 ± 0.08	0.002
Whole model	Adjusted *R*^2^ = 0.44	<0.001	Adjusted *R*^2^ = 0.70	<0.001

CTx: chemotherapy; ISS: International Staging System for multiple myeloma; PD: progressive disease; SD: stable disease; SMM: smoldering myeloma; uNGAL: urine neutrophil gelatinase-associated lipocalin.

**Table 2:** Demographic and clinical characteristics of patients at baseline.

Characteristic	MM patients (*n* = 73)
Mean age ± standard deviation, years	69 ± 10
Male sex, *n* (%)	38 (52)
Median time since diagnosis of MM (lower; upper quartile), months	36 (17; 69)
Smoldering myeloma, *n* (%)	6 (8)
ISS stage I, *n* (%)	40 (55)
ISS stage II, *n* (%)	15 (21)
ISS stage III, *n* (%)	12 (16)
Immunophenotype	
IgG, *n* (%)	52 (71)
IgA, *n* (%)	17 (23)
IgM, *n* (%)	1 (1)
Biclonal, *n* (%)	2 (3)
Free light chains only, *n* (%)	1 (1)
Nonsecretory, *n* (%)	3 (4)
Disease state on the day of study visit	
CR, *n* (%)	22 (30)
PR, *n* (%)	30 (41)
SD, *n* (%)	6 (8)
PD, *n* (%)	15 (21)
Chemotherapy on the day of study visit	
On (maintenance) treatment, *n* (%)	30 (41)
No treatment, *n* (%)	43 (59)
Number of prior treatment schemes	
No treatment, *n* (%)	8 (11)
1, *n* (%)	17 (23)
2, *n* (%)	22 (30)
3 and more, *n* (%)	26 (36)
History of auto-PBSCT, *n* (%)	28 (38)
Bone lesions, *n* (%)	44 (60)
History of acute kidney injury, *n* (%)	6 (8)

CR: complete remission; Ig: immunoglobulin; ISS: International Staging System for multiple myeloma; MM: multiple myeloma; *n*: number of patients; PBSCT: peripheral blood stem cell transplant; PD: progressive disease; PR: partial remission; SD: stable disease.

**Table 3:** Laboratory data of patients at baseline.

Characteristic	MM patients (*n* = 73)
Serum creatinine, *μ*mol/l	89 (76; 104)
eGFR (MDRD), ml/min/1.73m^2^	65.4 (49.6; 73.4)
>60 ml/min/1.73m^2^, *n* (%)	47 (64)
30-60 ml/min/1.73m^2^, *n* (%)	17 (23)
<30 ml/min/1.73m^2^, *n* (%)	9 (12)
Hemoglobin, g/dl	12.5 ± 1.8
Anemia (hemoglobin below lower reference limit), *n* (%)	14 (19)
Serum iron, *μ*mol/l	15.3 ± 4.9
Serum lactate dehydrogenase, U/l	360 ± 74
Lactate dehydrogenase above higher reference limit, *n* (%)	5 (7)
Serum *β*2-microglobulin, mg/l	2.75 (2.17; 4.20)
Serum albumin, g/l	41.7 ± 4.5
Serum-free light chains	
*κ*, mg/l	20.1 (13.0; 52.8)
*λ*, mg/l	19.1 (13.5; 34.3)
Involved serum-free light chains, mg/l	38.3 (17.4; 106.0)
Urine light chains	
*κ*, mg/l	ND (ND; 30.6)
*λ*, mg/l	ND (ND; 7.6)
Involved urine light chains, mg/l	7.8 (ND; 39.0)
NT-proBNP, pg/ml	74.8 (31.6; 287.2)

Data are shown as median (lower; upper quartile) or mean ± standard deviation unless otherwise specified. eGFR: estimated glomerular filtration rate; MDRD: Modification of Diet in Renal Disease; MM: multiple myeloma; ND: nondetectable: NT-proBNP: N-terminal pro-B-type natriuretic peptide.

**Table 4:** Laboratory results in MM patients as compared to control subjects.

Test	MM patients (*n* = 73)	Controls (*n* = 21)	*p* value
GDF-15, pg/ml	1259 (863; 1934)	584 (516; 762)	<0.001
Interleukin 6, pg/ml	2.97 (1.61; 6.00)	0.58 (0.19; 1.13)	<0.001
Hepcidin 25, ng/ml	28.8 (16.5; 44.6)	27.1 (20.0; 37.3)	0.9
Serum cystatin C, ng/ml	866 (689; 1287)	963 (874; 1038)	0.5
Urine cystatin C, ng/ml	56.0 (16.3; 105.5)	46.7 (26.5; 64.3)	0.5
Urine NGAL monomer, ng/ml	12.9 (5.7; 29.1)	8.7 (4.8; 11.0)	0.08

Data are shown as median (lower; upper quartile). GDF-15: growth differentiation factor 15; MM: multiple myeloma; NGAL: neutrophil gelatinase-associated lipocalin.

**Table 5:** Simple and multiple Cox proportional hazard regression models to predict the two-year overall survival of the studied patients with MM.

Independent variable	Simple regression		Multiple regression	
	Hazard ratio (95% confidence interval)	*p* value	Hazard ratio (95% confidence interval)	*p* value
ISS II or III	5.53 (1.76-17.39)	0.003	9.12 (2.05-40.72)	0.004
On CTx treatment	13.02 (2.93-57.95)	<0.001	15.02 (3.07-73.65)	<0.001
ln (GDF-15)	1.99 (0.97-4.08)	0.061	0.36 (0.12-1.04)	0.060

CTx: chemotherapy; GDF-15: growth differentiation factor 15; ISS: International Staging System for multiple myeloma.

## Data Availability

The data used to support the findings of this study are included within the article.

## Supplementary Materials

Supplementary Materials Table S1 details treatment among the studied sample of MM patients. Figure S1 is a graphical representation on the relationship between GDF-15 concentrations and different drug choice. Table S2 provides analyses for several studied biomarkers (variables) compared based on treatment status in MM patients.
