# Supplementary material for: Evaluating the Relationship of GDF-15 with Clinical Characteristics, Cardinal Features, and Survival in Multiple Myeloma
Source: Mediators Inflamm. 2020 Oct 21;2020:5657864. doi: 10.1155/2020/5657864 (PMC7596430; doi:10.1155/2020/5657864)
Supplement: Supplementary Materials — Table S1 details treatment among the studied sample of MM patients. Figure S1 is a graphical representation on the relationship between GDF-15 concentrations and different drug choice. Table S2 provides analyses for several studied biomarkers (variables) compared based on treatment status in MM patients. [file 5657864.f1.docx]

**Supplementary data**

**Table S1.** Treatment in studied patients with MM (n=73)

| **Therapy** | \| **n** \| \| --- \| | \| **Percent** \| \| --- \| |
| --- | --- | --- | --- | --- |
| untreated | 43 | 58.9 |
| Drugs: |  |  |
| dexamethasone (D) | 29 | 39.7 |
| bortezomib (V) | 10 | 13.7 |
| lenalidomide (R) | 13 | 17.8 |
| thalidomide (T) | 5 | 6.8 |
| cyclophosphamide (C) | 4 | 5.5 |
| melphalan (M) | 1 | 1.4 |
| Schemes: |  |  |
| \| RD \| \| --- \| | 12 | 16.4 |
| VD | 6 | 8.2 |
| \| CTD \| \| --- \| | 2 | 2.7 |
| \| VTD \| \| --- \| | 2 | 2.7 |
| \| T \| \| --- \| | 2 | 2.7 |
| \| VR \| \| --- \| | 1 | 1.4 |
| TD | 1 | 1.4 |
| \| MD \| \| --- \| | 1 | 1.4 |
| \| CD \| \| --- \| | 1 | 1.4 |
| \| D \| \| --- \| | 1 | 1.4 |
| \| VCD \| \| --- \| | 1 | 1.4 |


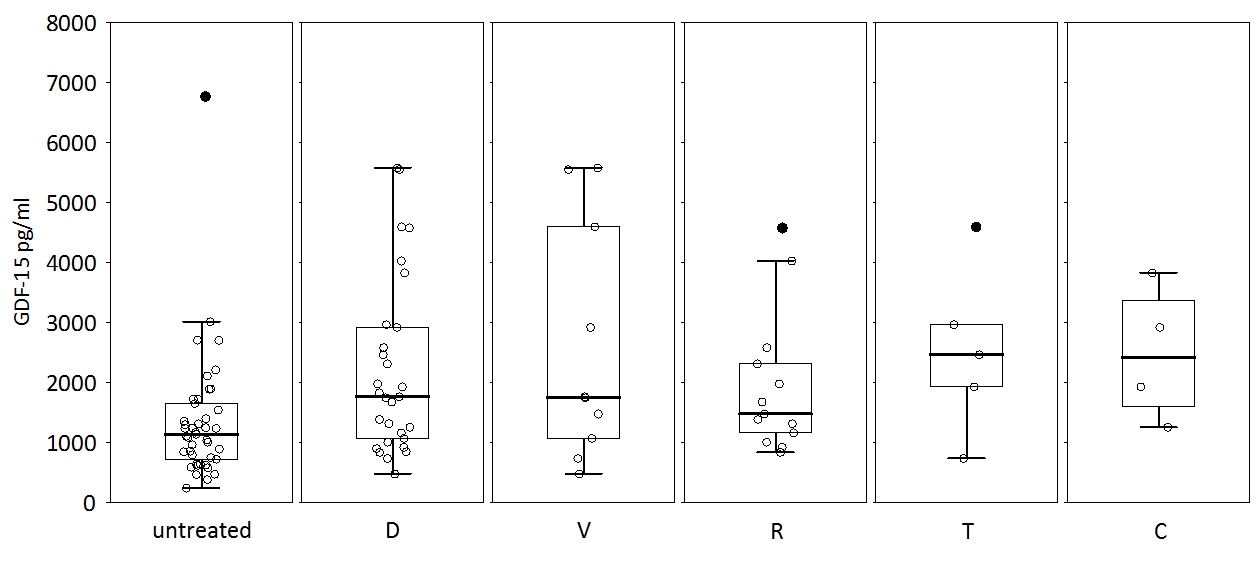


**Figure S1.** GDF concentrations among MM patients according to drugs used (the abbreviations are explained in Table S1). GDF-15 concentrations in untreated patients are shown for comparison. Data are shown as median, interquartile range (box), non-outlier range (whiskers) and raw values (circles).

**Table S2.** Correlations of GDF-15 with other studied variables among MM patients who received treatment at the start of the study (n=30) and in those who did not (n=43). Spearman rank correlation coefficient has been calculated as GDF-15 values were non-normally distributed.

| **Variable** | **MM patients on treatment** | | **Non-treated MM patients** | |
| --- | --- | --- | --- | --- |
|  | R | p | R | p |
| involved serum FLC | -0.07 | 0.7 | 0.38 | 0.012 |
| involved urinary LC | -0.01 | 0.9 | 0.41 | 0.006 |
| β2-microglobulin | 0.68 | <0.001 | 0.55 | <0.001 |
| albumin | -0.44 | 0.016 | -0.38 | 0.012 |
| blood hemoglobin | -0.54 | 0.002 | -0.42 | 0.005 |
| serum iron | -0.45 | 0.024 | -0.20 | 0.2 |
| hepcidin-25 | 0.19 | 0.3 | 0.11 | 0.5 |
| serum creatinine | 0.60 | <0.001 | 0.52 | <0.001 |
| eGFR (MDRD) | -0.63 | <0.001 | -0.43 | 0.004 |
| serum cystatin C | 0.80 | <0.001 | 0.59 | <0.001 |
| urinary NGAL | 0.77 | <0.001 | 0.38 | 0.013 |
| interleukin 6 | 0.22 | 0.3 | 0.35 | 0.021 |

eGFR, estimated glomerular filtration rate; FLC, free light chains; LC, light chains; MDRD, Modification of Diet in Renal Disease; MM, multiple myeloma; NGAL, neutrophil gelatinase associated lipocalin
